# 

**Published:** 1891-03

**Authors:** 


CONTENTS—MARCH, 1891.—VOL. VI., NO. 9.

ORIGINAL ARTICLES—
Transplantation of Tissue from Lower Animals to Man: Illustrated: A. M. Phelps, M. D., etc .............. 373
Carcinoma of the Stomach: A. Garwood, M. D........ 384
Cæsarean Section: Reported by Student Seth Morris, of Austin .......................................... 387

EDITORIAL—
Homeopathy and Homeopaths under the Calcium Light —Their Claims to Consideration in Medical Legislation ........................................ 389
Senators in Motley .................................. 398
Dastardly Outrage upon a Physician................. 401
A Card: F. E. Daniel................................ 403
Hope Deferred ....................................... 404
Dr. Lloyd's Loss by Fire ............................ 405

SOCIETY NOTES—
Austin District Medical Society—Proceedings and Resolutions ........................................ 406
The Cherokee County Medical Society................ 407
West Texas District Medical Society................. 408
Panhandle Medical Society .......................... 409
Mississippi Valley Medical Association............... 410
National Association Railway Surgeons .............. 410
Texas State Medical Association—Section on Practice . 410
Waco Meeting T. S. M. A., etc ...................... 410

MEDICAL NEWS AND MISCELLANY—
Numerous items of news of interest to physicians...... 412

PUBLISHERS' NOTES—
Having reference to advertisements, etc ............... 414

BOOK NOTICES......................................... 418

